# Vaccine hesitancy was not shown to be associated with traffic safety or driver behavior

**DOI:** 10.3389/fpubh.2023.1204205

**Published:** 2023-10-05

**Authors:** Jeff Wilson, Claus Rinner, Anna Pefoyo Kone, Edward Bosveld

**Affiliations:** ^1^Novometrix Research Inc., Moffat, ON, Canada; ^2^Department of Geography and Environmental Studies, Toronto Metropolitan University, Toronto, ON, Canada; ^3^Department of Health Sciences, Lakehead University, Thunder Bay, ON, Canada; ^4^Department of Politics and International Studies, Redeemer University, Hamilton, ON, Canada

**Keywords:** accident risk, confounding factors, COVID-19, prejudice, research ethics, social determinants, study design, vaccine hesitancy

## 1. Introduction

The study by Redelmeier et al. (1) generated significant news media echo in Canada with headlines such as “Unvaccinated drivers more likely to be in a car crash, study” (Globe and Mail, 22 Dec 2022), “COVID vaccine refusers have 72 percent higher risk of a serious traffic crash, study shows” (Vice, 13 Dec 2022), or “Auto insurers may charge unvaccinated drivers more for insurance—report” (Insurance Business Canada, 19 Dec 2022). Indeed, traffic accidents create serious harms to individuals and communities, so their contributing factors warrant further investigation. The authors provide a hypothesis-generating observational study that endeavors to evaluate a potential link between involvement in a traffic accident and COVID-19 vaccine hesitancy in the province of Ontario, Canada.

Complex public health issues and effective policy interventions benefit from consideration of multi-disciplinary perspectives. To that end, we offer here a perspective from public health epidemiology and outbreak response. We find serious flaws in Redelmeier et al.'s study design, variable selection, and group characterizations.

## 2. Study design and framework not suitable to test stated hypothesis and research question

The authors' hypothesis is “that individual adults who tend to resist public health recommendations might also neglect basic road safety guidelines”, and they ask: “Does COVID vaccine hesitancy correlate with the risks of a serious traffic crash?” However, no data were collected about neglect of road safety nor about vaccine hesitancy.

The authors incorrectly imply that anyone involved in a traffic crash in this study had neglected road safety guidelines, however, many of the persons included in their dataset may in fact have been victims rather than at fault. This is especially likely to be the case for passengers.

Similarly, the authors wrongly assume that anyone who remained unvaccinated by July 2021 was vaccine hesitant. Instead, individuals may have declined or delayed vaccination for reasons such as recent COVID-19 infection, pregnancy, allergy to vaccine ingredients, or other medical concerns. Conversely, it should not be assumed that there were no vaccine hesitant individuals among those vaccinated by July 2021. Many Ontarians–including those who were vaccine hesitant–were required to and did receive the COVID-19 vaccine as a condition of employment or to travel. Others may have received the vaccine following pressure from friends, family, health providers or government, despite their hesitancy. Indeed, the authors acknowledge that “the study does not test the reliability of COVID vaccination as a proxy for COVID vaccine hesitancy”.

If individuals who were not at fault were excluded from the traffic accident dataset and vaccine hesitancy had been correctly measured, what impact might this have had on the results of this study?

## 3. Important factors influencing vaccination status and likelihood of being involved in a traffic accident were not considered

The study seeks to calculate risk by comparing the proportion of unvaccinated individuals involved in traffic crashes with their proportion in the general population; however, it fails to consider whether unvaccinated individuals were more likely to be traveling and thus over-represented among drivers, passengers, and pedestrians. Data regarding several important variables were not included in this study. These include distance traveled, occupation, education and immigration status. In the Discussion section, the authors appear to dismiss “distance driven” as a potential confounder, citing a prior study by the lead author (2). That study, however, casts doubt on this conclusion, stating “traffic risks increase if motorists travel longer distances.” It is also worth noting that increased driving distance is associated with driver fatigue, a leading cause of fatal crashes (3). Drivers–particularly delivery and truck drivers–have experienced increased exhaustion during the pandemic due to increased demand and challenges (4). Finally, it is distance traveled that should have been controlled for rather than distance driven, since pedestrians were included in the study.

Individuals from certain “education levels, industries and regions” were found to be substantially less able to work from home during the pandemic and were therefore more likely to be commuting (5). Vulnerable workers in the gig economy, including those driving for ride-hailing and delivery services, were considered essential workers and therefore may have shared an increased burden of traffic risks during the pandemic (6). This higher risk may have affected recent immigrants who are disproportionately represented among gig workers (7).

Are similar factors also related to vaccination status? Indeed, they are. Data collected during the same period as Redelmeier et al.'s (1) study show that Canadians with less than secondary education (16%) were more than twice as likely to be unvaccinated as those with a university education (7%) (8). This may be explained in part by the fact that vaccination policies or mandates in Canada have varied by employment sector (9). Data from 2021 also show that rates of COVID-19 vaccination were lower among immigrants, refugees, and recent OHIP registrants (who were more likely to be gig workers) compared to Canadian-born or long-term residents (10).

Did the authors consider the baseline rate of vaccination among those who were more likely to be traveling during the pandemic? Is it possible that occupational requirements to travel in conjunction with demographic characteristics, rather than vaccine hesitancy, are responsible for accident risks?

## 4. Assumptions about unvaccinated individuals may lead to discrimination against certain socio-demographic groups or racial backgrounds

The study hypothesis assumes that unvaccinated individuals are a homogeneous group in that they “resist public health recommendations”. However, it cannot be assumed that individuals who were not (or not yet) vaccinated by July 2021 fail to comply with public health advice as a general rule. For example, data from 2021 show that Canadians who had not received the COVID-19 vaccine were less likely to be regular alcohol users than those who were vaccinated, suggesting that those who were unvaccinated were more likely to comply with public health guidance regarding alcohol use (11).

Is it reasonable to assume, as the study hypothesis suggests, that Ontarians who do not comply with public health advice regarding fruit and vegetable consumption or physical activity are at greater risk of a traffic crash?

Unvaccinated Canadians are a diverse group of individuals: coverage rates vary by education and immigration status (as described above), income, and ethnic identity. In 2021, Canadians with an annual household income of <$30,000 (7%) were significantly more likely to indicate that they would not get vaccinated compared to those from the highest income group ($150,000 or more; 3%) (12). Government data also show that vaccination coverage has been lower among individuals who self-identify as off-reserve First Nation (81%), Black (82%) or Arab (85%) compared to those who do not identify as a visible minority or Indigenous (93%) (13).

Is it appropriate to suggest that differences observed in this study might “justify changes to driver insurance policies in the future”, when data clearly show that vaccination status differs by factors associated with socio-economic status, including education, income and ethnic identity?

## 5. Conclusion

The news media uptake of Redelmeier et al. (1) shows the potential real-world impacts of public health research–for example, increased stigma and higher auto insurance fees, which could disproportionately impact certain marginalized groups. In this response, however, we have outlined several concerns that cast serious doubt on the conclusions and recommendations offered by both the study authors and the media. We urge the authors (and other researchers with access to the same or similar population-level datasets) to conduct a re-analysis of the data, with the addition of the control variables mentioned above; this could address statistical issues like those identified by Ioannidis (14). Scientists, peer reviewers, and journal editors must work responsibly to avoid real or perceived bias that may lead to erroneous findings and potentially harmful conclusions [e.g., (15)]. Even in the hectic environment of a developing pandemic, we need to follow established research methods and ethical principles that have helped us weather many previous public health crises.

## Author contributions

All authors contributed equally to the conception, drafting, and revision of this opinion.
